# Healthcare and social care professionals’ experiences of respite care: a critical incident study

**DOI:** 10.1080/17482631.2024.2352888

**Published:** 2024-05-12

**Authors:** Annelie K. Gusdal, Mirkka Söderman, Tina Pettersson, Jaana Kaup, Lena-Karin Gustafsson

**Affiliations:** School of Health, Care and Social Welfare, Mälardalen University, Eskilstuna/Västerås, Sweden

**Keywords:** Critical incident technique, informal care, group interviews, older adults, professional caregivers, qualitative research, respite care

## Abstract

**Introduction:**

Aging in place is favoured among older persons and supported by research in Sweden, although it poses challenges for overburdened informal caregivers. While respite care can offer support, its accessibility is hindered by organizational challenges and informal caregivers’ delays in using it. The experiences of informal caregivers are well-studied, but the professionals’ experiences of respite care quality and critical incident management are underexplored.

**Aim:**

To explore professionals’ experiences of critical incidents in respite care, consequences for the persons being cared for, and strategies to manage critical incidents.

**Materials and methods:**

A qualitative, critical incident technique was used, and three group interviews with a total of 16 professionals were conducted.

**Results:**

Barriers to quality respite care included communication gaps during care transitions, environmental shortcomings in respite care facilities, lack of support for informal caregivers, and inadequacies in respite care decisions. Strategies to manage critical incidents included individualized care, continuity and communication in care transitions, a conducive environment, support for informal caregivers, and care professionals’ positive approach.

**Conclusions:**

The study emphasizes the need for focused efforts on communication, continuity, and a supportive environment. Addressing identified challenges and applying suggested strategies will be key to maximizing the potential of respite care as a vital support for care recipients and their informal caregivers.

## Introduction

An increasing number of older persons have the privilege of residing in their own homes, with the desire and ability to maintain their independence and exercise control over their daily lives (Gusdal et al., 2021). The aspiration for independence among older persons with cognitive impairment or functional limitations should not be dismissed. A growing body of research advocates “aging in place”, which implies that its advantages surpass its drawbacks, if the older person’s health and social care needs are met. Providing the requisite conditions for older persons to stay in their homes as long as possible supports not only their active lifestyles but also their independence (Sixsmith et al., 2014). Preventive and supportive work is thus focused on maintaining independence and on delaying age-related healthcare and social care needs (Bloom et al., 2015; WHO, 2017). Conversely, the aspirations for an active and independent life while ageing in place must be balanced against the specific needs of informal caregivers who care and support older persons with cognitive impairments or functional limitations. Informal caregivers, despite their resourcefulness, often remain unrecognized or undervalued and are commonly depicted as overwhelmed and unprepared. As older persons prefer to age in place (Rantz et al., 2014), integrated respite services are vital for ageing in place with or without home care.

Given that informal caregiving is emotionally and physically demanding, and informal caregivers require time to rest and recuperate from their caring roles, a range of community health and social care services are available, including home-based interventions and/or respite care outside the home (Moholt et al., 2020). Respite care is a temporary care service that provides relief to informal caregivers, allowing them to take a break from their caregiving responsibilities. It is often used by families caring for loved ones with special needs, disabilities, or chronic illnesses. Respite care can be provided in various settings, such as in-home care, day centres, or residential facilities, and can range from a few hours to several days or weeks (Rao et al., 2021). In the current article respite care refers to the care given in residential facilities.

The primary precondition for informal caregivers’ positive experience of respite care is that they perceive the benefit of the service (Timmons et al., 2019). Previous research from the perspective of healthcare and social care professionals is notably sparse but in the existing few studies professionals contended that it is challenging for informal caregivers to access respite care and argued that the service should be less bureaucratic and more easily accessible for families in need of support (O’Shea et al., 2017). The complexities of the healthcare system make it difficult for informal caregivers to navigate, thus necessitating simplified access to and information about available support services (O’Shea et al., 2019). Organizational constraints, including staffing shortages, time restrictions, and financial limitations, act as significant impediments to the expansion of respite care and compromise the delivery of high-quality services (E. O’S. Ohea et al., 2017).

Professionals struggle to understand why informal caregivers do not seek respite care despite a clear need or tend to delay seeking help until it is almost too late (O’Shea et al., 2017). The reasons can be attributed to a combination of factors including informal caregivers’ lack of knowledge, difficulty navigating the healthcare system (Bragstad et al., 2014), lack of trust in formal health care services (Groenvynck et al., 2022) and the emotional and physical demands of caregiving (Groenvynck et al., 2022; Roberts & Struckmeyer, 2018).

The growing trend of ageing in place has intensified the need for respite care services, which are sometimes hindered by bureaucratic and resource constraints. While research has focused on the experiences of informal caregivers, there is a notable gap in understanding the perspectives of professionals, who face challenges in delivering quality respite care and managing critical incidents. Given this context, the study aims to explore professionals’ experiences of critical incidents in respite care, consequences for the persons being cared for, and strategies to manage critical incidents, thereby contributing valuable insights for enhancing respite care quality.

## Materials and methods

### Design

The study has a qualitative descriptive design with an inductive approach using group interviews (GIs) for data collection inspired by Holloway and Wheeler (2010), Krueger and Casey (2015), and McLafferty (2004). The interviewing was based on the Critical Incident Technique (CIT), focusing on variations in perceived critical incidents. An incident can be understood as a dilemma, episode, event, or situation in the persons’ lives (Flanagan, 1954). The five-step analysis process is applicable across various health and welfare disciplines and has been developed within nursing to visualize both experiences and care interventions.

### Study setting, participants and recruitment procedure

The study was conducted in one urban municipality in the mid-east of Sweden from August to November 2022. The municipalities in Sweden have a mandatory commitment to provide health and social care for disabled persons and those >65 years in ordinary homes and nursing homes that provide respite care. Participants of interest for this study were healthcare and social care professionals working within respite care services, persons holding professional support roles for informal caregivers, and social workers involved in the assessment process for respite care eligibility. Temporary and untrained staff did not meet the inclusion criteria and were excluded from the study. Contact information for prospective participants for the study was obtained from developers within the Health and Social Care Administration in the municipality. A total of 33 professionals were provided with information about the study via email and invited to participate. Of these, 16 agreed to partake in the study and were extended invitations for interviews and a time and place for the GI was agreed upon. Thereafter, written information on the study and a written form of informed consent were sent to the professionals.

### Data collection

With the intention of exploring in-depth and wide-ranging experiences and perceptions, GIs were used. This mode of data collection is shown to elicit rich information (Krueger & Casey, 2015). Three GIs with a total of 16 participants were performed with 4–7 participants in each interview group (Holloway & Wheeler, 2010; Krueger & Casey, 2015). For practical reasons the GIs were conducted in the participants’ respective workplaces. Participants in each GI were acquainted with each other and, in some instances, were colleagues within the same respite care facility. Thus, an intra-group homogeneity was achieved (McLafferty, 2004).

The GIs were moderated by one of the authors (JK or TP), a research assistant and lecturer with experience and training as a moderator of GIs. A semi-structured interview guide was used with open-ended questions, adding follow-up questions and probes when needed (Krueger & Casey, 2015; Kvale & Brinkmann, 2009). The interview guide was developed by the research group inspired by the literature of respite care (O’Shea et al., 2019; O’Shea et al., 2017; Schulz et al., 2020). The interview questions used in the GIs are included in an appendix. The moderator balanced the GIs so that all questions in the interview guide were evoked. An assistant moderator (JK or TP), a research assistant and lecturer with experience of GIs, took observational notes and helped to recapture and summarize points of relevance to the study aims at the end of each GI. The participants then reflected, verified and further developed the content, a recommended method of validation of GIs (Krueger & Casey, 2015). The interviews lasted 36–56 minutes and were digitally audio recorded. A summary based on observational notes and a debriefing session were performed by the moderator and the assistant moderator immediately after each interview. The interviews were transcribed verbatim in its entirety and the transcriptions were verified by the moderator, assistant moderator and the first author.

### Data analysis

Data from the GIs were analysed using CIT (Flanagan, 1954), described by Svensson and Fridlund (2008) and using QSR NVivo 1.7.1 software program (2022). The five-step analysis process was conducted as follows:

1. The three digitally audio recorded GIs were listened to in order to gain an overarching understanding of the content, after which they were transcribed verbatim. The transcribed text was then read through carefully multiple times to become familiar with the content.
2. Critical incidents consistent with the study aims were identified.
3. Critical incidents with similar content were coded/labelled in a structural analysis table using the NVivo software program and were classified into subcategories.
4. Subcategories with similar content were coded/labelled and sorted into categories.
5. Categories were coded/labelled and organized into main areas.

The analysis process was discussed within the research team to achieve a high level of credibility, particularly with respect to the confirmability of the analysis process.

### Ethical guidelines

The participants were informed that their participation was voluntary and that they could end their participation whenever they wished without providing an explanation. Written informed consent was obtained prior to conducting the group interviews. The study was approved by the Ethical Review Authority in Sweden (Dno 2022–01835–01) and conforms to the principles outlined in the Declaration of Helsinki (World Medical Association, 2018) and informed consent was obtained from all participants in accordance with the European Union’s General Data Protection Regulation [GDPR] (European Parliament and Council of the European Union, 2016/679).

## Results

### Participants

The first GI consisted of nursing assistants (*n* = 4) with professional experience ranging from 8 to 41 years. The second GI consisted of four registered nurses, one district nurse, and two specialized nursing assistants with a focus on dementia care (also known as “Silvia sister”) (*n* = 7), with professional experience ranging from 21 to 30 years. The third GI consisted of one nursing assistant, one behavioural scientist, two social workers, and one physical therapist (*n* = 5), with professional experience ranging from 5 to 16 years. All participants were female, with a median age of 51 years and a median professional tenure of 21 years (Table 1).

**Table 1.** Characteristics of the participants (*n* = 16) in the group interviews (*n* = 3).

Sex, female/male	16/0
**Age**, median (range)	51 (35–63)
**Profession**	
Nursing assistant	5
Specialized nursing assistant	2
Registered nurse	4
District nurse	1
Behavioural scientist	1
Social worker	2
Physical therapist	1
**Years in profession**, median (range)	21 (5–41)

In the analysis of the GIs, two main areas emerged. In the first main area, Barriers to quality respite care, four categories emerged addressing the aim: To explore professionals’ experiences of critical incidents in respite care and their consequences for the persons being cared for.

In the second main area, Strategies for achieving quality respite care, five categories emerged addressing the aim: To explore professionals’ experiences of strategies to manage critical incidents.

### Barriers to quality respite care

The main area (Table 2) encompasses four categories: Deficiencies in communication and continuity during care transitions, Shortcomings in indoor and outdoor environments, Lack of recuperation, support, and assurance for informal caregivers, and Inadequacies in decisions about respite care—not the appropriate type of care.

**Table 2.** Summary of the main areas, categories, and subcategories of critical incidents.

Main area	Category	Subcategory
		Deficiencies in communication between the respite care facility and the person’s home/home care service and primary healthcare centre (7)
	Deficiencies in communication and continuity during care transitions	Deficiencies in continuity between the respite care facility and day care activities (4)
		Unsettled environment (14)
	Shortcomings in indoor and outdoor environments	Drab and confined environment (4)
Barriers to quality respite care		Professionals contacted the informal caregivers for consultation (10)
	Lack of recuperation, security, and support for informal caregivers	Informal caregivers’ guilt for placing the person in a respite care facility (7)
		Informal caregivers’ mismanagement of the person’s medication and/or wound care at home (3) Informal caregivers’ limited knowledge on how to support the person’s self-care capability (4)
		Admittance to respite care facility at a very late stage (8)
	Inadequacies in decisions about respite care - not the appropriate type of care	Financial incentives (6)
		Not individualized respite care decisions (18)

Number of critical incidents in parentheses.

Subcategories under each category are written in ***bold, italicized*** text, and the numbers in parentheses correspond to the number of critical incidents in the respective subcategory.

#### Deficiencies in communication and continuity during care transitions

Participants described the dilemma of providing quality respite care while struggling with prevalent deficiencies in communication and continuity in diverse care transitions.

***Deficiencies in communication between the respite care facility and the person’s home/home care service and primary healthcare centre*** (7) resulted in logistical issues such as bringing incorrect clothing or belongings for the person upon departure from home, or the professionals’ unpreparedness regarding the person’s condition upon arrival at the facility. This could lead to delays in physiotherapists’ planning schedules and ambiguities about who was responsible for the person’s exercise program.

We are very rarely contacted. And it feels strange. Because it seems like this is a group that should have a need for both a lot of exercise and a lot of help with their transfers. And then it also becomes this thing that, if one calls us at the end of the week, we won’t be able to make it there that week … it perhaps requires a bit more planning.//No, it occurred to me that they might not have home care services. And the husband or wife helps them at home and then they are supposed to get help from the staff. And then it becomes a bit difficult. Who oversees the exercise program? Because then it’s not really us, and it’s not really anyone. (P. 16)

***Deficiencies in continuity between the respite care facility and day care activities*** (4) meant that persons could not attend their regular day care activities and therefore lost their routine when they were at the respite care facility.

And it has indeed been a dilemma, it’s what we have been advocating for and highlighting—that it’s a problem when one cannot maintain that continuity. I am thinking about the man who attended the day activity centre … and then disappeared from the respite care. He was knocking on the door of the day activity centre, wanting to come in and have breakfast, and they did not have the opportunity to let him in because they had taken in another guest that day when he was supposed to be in respite care. And then it becomes difficult to tell someone who is used to entering there, that “no, you can’t be here this week, but you are welcome again next week”. (P. 11)

This deficiency in continuity also meant that the person had to interact with multiple professionals from both facilities, who did not always communicate effectively with each other.

#### Shortcomings in indoor and outdoor environments

Participants described the dilemma of providing quality respite care while struggling with the reality of working in ***unsettled environments*** (14) when there was a high turnover of persons in the respite care facility, as with rapid and temporary admissions. As a result, persons sometimes had to share rooms or stay in very impersonally decorated rooms.

We had to move all the time, and emergency placement (rapid and temporary admissions) came in, someone had set fire to their apartment and urgently needed a place, so it was resolved like, “oh well, you had a single room, now you have to share it because another one is coming …” (P. 7)

… the respite care facility became like a train station, it was quite chaotic there, with packing and moving in and out, and people being picked up; there was a substantial flow. They also mixed in emergency placements and everything, so it became no home at all. They have no personal belongings, just get a bed, a room. So, it’s very stripped-down where they live, it becomes very impersonal. (P. 5)

In such an unsettled environment, the persons did not only become anxious, but their informal caregivers also experienced a lack of security. For some persons, the environment became particularly unsettling when they could not be assigned the same room within the respite care facility.

… with the logistics, to piece it together, that someone was there for a week and some for two, and then someone got sick and didn’t come … yes, like that. (P. 5)

The environment was further unsettled when the professionals had constrained schedules that did not afford them the time to sit down and establish a sense of security or presence with newly arrived persons. Instead, a significant proportion of their time was spent on cleaning, laundry, and dishwashing—tasks that participants argued should be allocated to specialized cleaning or service personnel so that care professionals could focus on the well-being of the persons.

Participants also described deficiencies in terms of a ***drab and confined environment*** (4), which they likened to a prison setting where persons were constantly monitored and had restricted freedom of movement.

#### Lack of recuperation, security, and support for informal caregivers

The participants described the dilemma of providing quality respite care while struggling with a lack of recuperation, security, and support for informal caregivers. This was particularly apparent when the ***professionals contacted the informal caregivers for consultation*** (10) on managing the person’s varying preferences, such as whether the person wished to go outside, or opting out of personal hygiene routines such as showering or brushing their teeth.

That one can ask, “Your husband wants to go out; should we let him out or how should we handle it?”, “Will he come back if we open the door?”. Since you can’t, for example, lock them in. And “if he refuses to shower, what should we do then?”, “Can you come over here and help us brush his teeth?” (P. 11)

Because if she [the informal caregiver] says yes to a walk, then she’ll lie there thinking “what if he doesn’t come back” and like this … Well, then there’s no rest, then there’s a conscience that … (P. 10)

The participants also described how informal caregivers experienced a lack of recuperation, security, and support when they felt ***guilt for placing the person in a respite care facility*** (7). This sentiment was especially pronounced when the person expressed a preference to stay at home, but the informal caregiver felt overwhelmed by the demands, or when the person overtly displayed mistrust towards the informal caregiver.

And she has a great need to be alone, and there is a dilemma, that she feels guilt for leaving him behind, so that week she might get now, it may not be so good for her because she will feel guilty that he cannot stay at home. (P. 6)

The participants also reported that during the respite care stay, they observed ***informal caregivers’ mismanagement of the person’s medication and/or wound care at home*** (3). These observations were characterized as indicative of a lack of security and support for the informal caregivers.

… of course, there are certain medical situations, where you might discover that … there’s responsibility for medication, maybe someone else in the home environment needs to take over that, like home care services or something … wounds, or the risk of wounds, that need to be monitored in some way. I can imagine that these are the kinds of issues that are identified in a better way when there are experienced care professionals and access to a nurse. (P. 8)

The participants described a lack of support for informal caregivers, attributing this to their ***limited knowledge on how to support the person’s self-care capability*** (4). Informal caregivers were described as overly protective, thereby hindering, or depriving the person of their self-care abilities.

I usually think this way, if they can dress themselves, what does it matter if they make a mistake now and then? They need to feel that “I can do it”. But then there are informal caregivers who take away a lot from them … (P. 2)

#### Inadequacies in decisions about respite care—not the appropriate type of care

The participants described the dilemma of providing quality respite care while a permanent nursing home would have been a more appropriate option for persons who were ***admitted to the respite care facility at a very late stage*** (8). At this late stage, they persons were unable to utilize the resources of the respite care facility as they were too ill.

… one thought, “good heavens, why is he here?” And he’s so ill the first time he arrives. Because I thought that, yes, I didn’t believe he would survive. (P. 2)

Respite care at a late stage generated confusion and anxiety for persons with advanced dementia, as well as for their informal caregivers. For them, the late-stage respite care also meant that they had struggled excessively long, and the boundaries of what they could endure had been pushed forward, which was described by the participants as highly strenuous for the informal caregivers.

***Financial incentives*** (6) for choosing respite care over a permanent nursing home care were described from both an organizational perspective and from the perspective of the informal caregivers. The cost for the municipality was found to be lower when persons resided in respite care facilities compared to permanent nursing homes. This cost-benefit was similarly observed for the persons themselves and their informal caregivers.

It’s quite common, I’ve heard from many, with rent and … I mean, the maximum fee, food, and everything, it comes to 13,000 crowns [Swedish currency]. Yes, and then a home on top of that … for the informal caregivers … (P. 7)

So, it’s this thing about feeling guilty (as an informal caregivers) but also the financial aspect, that one chooses not to have that extra cost. Then maybe it results in seven years of respite care. (P. 11)

The participants also described that in some instances, ***decisions about respite care were not individualized*** (18) to the specific needs of the person and their informal caregivers. Such decisions were often instituted on an “until further notice” basis rather than undergoing periodic evaluations, such as semi-annually or annually. This approach had implications for both the person and their informal caregivers. Frequent transitions, over a longer period, posed a potential of the person feeling a lack of belonging, both in the respite care facility and in their own home.

… even younger persons who might still have informal caregivers who work, so then one week you’re in respite care, the next week you’re in daily activities (whilst living at home), the next week you’re in respite care, so you never really feel like you’re at home. (P. 7)

And many of the persons we’ve met say that “I’m always on the move”, because some wives say that “I can’t be bothered to unpack this bag, so it stands ready-packed in the hallway”, so even when they’re at home, they are reminded that next week they will have to leave again. (P. 11)

The participants described potential challenges faced by persons who, although receiving extensive care at home from an informal caregiver—encompassing bathing, dressing, and assistance with eating—found that such care and support was not part of the home care service decision. Thus, these services were unavailable at the respite care facility. However, there was divergence among the participants regarding how these care service decisions were, and should be, handled at the respite care facility.

… a bit with routines and workplace culture, in the respite care they’ve always worked from the perspective of not looking at care assistance decisions but offering an all-inclusive care, which is also wrong because your care should be based on needs. (P 6)

The person could receive more help at the respite care facility than they previously did at home with the combined support of informal caregivers and home care services. While this enhanced level of care might create a sense of security for the person, it could inadvertently induce stress in the informal caregiver before the person’s impending return home.

I feel that the person can feel safe with more help than they get at home. So, if they get more help with their transfers, for example, it can feel safe. But it may not feel safe for the informal caregiver. Because then the informal caregiver knows that they come home in worse condition, and what they work for at home disappears when they are away. (P 16)

### Strategies for achieving quality respite care

The main area (Table 3) encompasses five categories of strategies for achieving quality respite care: An individualized care, Continuity and communication in care transitions, A good indoor and outdoor environment, Support and security for informal caregivers, and Care professionals’ internal and external resources.

**Table 3.** Summary of the main areas, categories and subcategories of critical incidents.

Main area	Category	Subcategory
		Create a personal environment (6)
	An individualized care	Tailor activities to the person’s habits from home (13)
		Remain in their home setting with increased home care services or offer an alternative to the respite care (16)
Strategies for achieving quality respite care		Informal caregiver and the person’s contact person accompany them into the respite care facility (14)
	Continuity and communication during care transitions	Continuity within the professional group (6)
		Create a homely environment (7)
	A good indoor and outdoor environment	Create an environment that facilitates companionship (6)
		Clear and open communication, coupled with high accessibility (24)
	Support and security for informal caregivers	Empathetic, understanding, and attentive approach (23)
		Commitment and engagement (17)
	Care professionals’ internal and external resources	Knowledge and competence (30)
		Time and organizational resources (8)

Number of critical incidents in parentheses.

Subcategories under each category are written in ***bold, italicized*** text, and the numbers in parentheses correspond to the number of critical incidents in the respective subcategory.

#### An individualized care

The participants described that a strategy to address an individualized care meant prioritizing individual needs through ***creating a personal environment*** (6). Creating this personal environment could be achieved through life narratives and by allowing persons to bring and store their personal belongings at the respite care facility. At one facility, there were lockable wardrobes where the person could store items such as paintings, blankets, stuffed animals, photographs, and a radio, and retrieve them during subsequent stays. This approach was suggested for all respite care facilities. Working with life narratives entailed engaging with reminiscence or memory prompts, which not only fostered security but also stimulated the person.

And I believe that both recognizing who I have been and who I am, as well as being familiar with the environment, like having photo albums or personal items that one is interested in. Even if I can’t knit or crochet anymore, it was once a major interest of mine, so provide some of the textiles I crafted, and we can at least sit and look at my past creations. It’s about always having individualized activities.//I believe that having something familiar, something to hold onto, can also instil a sense of security for those who are unwell … (P 11)

Another strategy to prioritize individual needs was to ***tailor activities to the person’s habits from home*** (13). This could be done, for instance, by allowing the person to sleep in, have breakfast in bed, stay up late, serve themselves food, participate in whatever is feasible—which was considered an activity—and ensure that care and attention mirrored what the person received at home.

It’s essential that we know their preferences, and that we are informed by their informal caregiver. Otherwise, the person might become very anxious when, in the morning, we say “come on, let’s go to the kitchen for breakfast”, “What? At home, I sleep till maybe nine or ten” … they become very upset and anxious. (P 3)

… that they’re involved in meals, that they get to spread butter on their own sandwiches.// … when they come to a facility, suddenly they can’t go into the kitchen, or take toppings themselves … they’re deprived of participating in their own life. (P 6)

They were also allowed to join in the kitchen and bake, if they adhered to the same hygiene standards as us, of course. (P 7)

Yet a strategy to address individual needs meant either allowing the persons to ***remain in their home setting with increased home care services or offer an alternative to the respite care*** (16), such as care in a dementia care facility where specialized expertise is available. Another suggested alternative was a temporary short-term stay, without a formal respite care decision, when informal caregivers needed a break to rest and recuperate for a few days. To ensure both the person and their informal caregivers feel safe and comfortable at home for as long as possible, tailored solutions are required. These include some form of relief for caregivers to ensure their long-term well-being.

…we also have those who might not fit into the respite care, so we opt to offer them care elsewhere, but they have the respite care decision while being where they need to be. Sometimes it’s beneficial to move to a dementia care facility with that decision, knowing that eventually, this person will need to relocate there, or that the right expertise is available to address the challenges. I think it’s good if we can think outside the box and collaborate. (P 6)

…but the one needing respite isn’t the person; it’s the informal caregivers. They need some form of support; they’re flexible. (P 8)

She should have a place where she can go and rest, a convalescent home where she can simply be without him constantly around, especially if relocating him is difficult. He might feel better in his home setting with much more care, while she gets to relax elsewhere and feels good. (P 10)

#### Continuity and communication during care transitions

The participants described that a strategy to address the dilemma of deficiencies in communication and continuity in care transitions involved having an ***informal caregiver and the person’s contact person accompany them into the respite care facility*** (14). They communicated with the professionals about the recent home situation and handed over belongings that were significant for the person’s stay—especially important if it was their first time at the facility. Similarly, the contact person would meet the person and their informal caregiver when leaving the respite care facility or upon arrival at home.

This communication, or handover, created a foundation for the security and comfort of both the person and their informal caregiver.

I believe it’s about security. I mean, that’s probably the crux of the matter. If the person feels safe, then perhaps their informal caregiver feels safe too, and vice versa. And then, as we’ve discussed, the handover process, especially when they have home care … so that it works well. (P 13)

The participants described that ideally, the same care professionals should accompany the person, as is the practice in the small municipality of Tibro, to enhance the sense of security for the person and their informal caregiver.

The needs are solely based on the person, so there they have home care professionals who accompany the person to day activities, and to respite care to handover the person to care professionals in the respite care facility. (P 5)

However, in the absence of this logistical possibility in the present municipality, the continuity of the contact person role remains, regardless of where the person is located, to enhance continuity and communication in care transitions between respite care facilities and the home. Alternatively, having a contact person at the respite care facility and another in home care, with both communicating with each other, was practiced.

I think more about the person here [at the respite care facility], getting more information about them can create context for those working here. Knowing what to talk to them about, like their interests, recent events, and what they’ve done before. I mean, it can create so many different things to talk about. (P 12)

I’m not sure what’s best—a phone call or a message of some sort. Because things happen here [at the respite care facility] too, which we need to know later at home. (P 15)

Besides the sense of security provided by the informal caregiver and the designated contact person; the participants described that ***continuity within the professional group*** (6) at the respite care facility presented a strategy to identify issues potentially overlooked in home care due to occasional high staff turnover. Continuity and security also increased for the person and their informal caregiver if the person continued attending their day activities even during their respite care stay.

#### A good indoor and outdoor environment

The participants described that a strategy to address the dilemma of shortcomings in indoor and outdoor environment meant ***creating a homely environment*** (7), and ***an environment that facilitated companionship*** (6) among those who wished to socialize. A homely environment was described as pleasant and the direct opposite of a stripped-down hospital environment, which could also calm persons with dementia; a homely environment increased comfort and participation in activities, such as in the kitchen.

The kitchen is open, and everyone comes in, and those who can, help themselves to some yogurt… it’s really good, really important. Those who can… I see it’s appreciated by some … (P 1)

An environment that facilitated companionship included access to an outdoor area where care professionals could grill or simply sit and have a coffee with the persons. Also, creating a “small room” using music or conversation in a smaller seating group within a larger room was also described to foster companionship.

#### Support and security for informal caregivers

The participants described that a strategy to address the dilemma of lack of support and security for informal caregivers meant having ***clear and open communication, coupled with high accessibility*** (24), which was integral to an informal caregiver’s perception of support and security. Informal caregivers needed assurance that their loved ones were well-cared for, safe, and part of a social context that enhanced their quality of life. Safety and security experienced by the persons in respite care often led to a comforted informal caregiver.

One could argue that for the informal caregiver, communication with the respite care facility is very important. It provides them with a sense of security, and they get the respite they need, to recuperate and to continue providing support as they do. (P 12)

Many informal caregivers struggled to understand or accept the reality, especially concerning dementia. Therefore, it was essential for care professionals to offer clear explanations and support informal caregivers in their journey of realization and acceptance.

Informal caregivers appreciated the opportunity to call whenever they had questions, felt anxious, or sought information about their loved one’s well-being, especially in the initial stages of the stay.

I work late, just go home, and relax. If there’s anything special, I’ll call you and tell. And you’re free to call anytime. We have a phone, you have a phone, call if you feel anxious or anything like that. And she was pleased … many say, “thank you for saying that … it was helpful.” There are informal caregivers who say, “it’s such a relief to let go.” (P 1)

Clear and open communication, coupled with high accessibility, implied that the care professionals dedicated time to engage with informal caregivers, especially regarding changes in the person’s condition or disease progression.

The participants described that a strategy also involved having an ***empathetic, understanding, and attentive approach*** (23) towards informal caregivers, which was critical for their experience of support and security. For informal caregivers, the emotional challenge lay in deciding to place their loved ones in respite care. They often struggled with letting go and could grapple with feelings of guilt, grief, anxiety, or exhaustion from continuous caregiving. Hence, offering support and allowing them a respite to reflect on their situation was vital. Informal caregivers also faced challenges in dealing with other informal members’ denial or lack of understanding of the circumstances.

But there have been several instances like this … when the wife felt overwhelmed and wanted to seek respite care, but then the children step in saying, “no he should remain at home”. And then you think, my God, they are not the ones staying at home with him. (P 2)

The participants underscored the importance of professionals being aware of these emotional dynamics for informal caregivers. By being attentive, supporting informal caregivers in understanding the situation, and assisting them in decision-making, trust was fostered between the care professionals and the informal caregivers.

#### Care professionals’ internal and external resources

The participants described care professionals’ ***commitment and engagement*** of (17) as a strategy, which occasionally extended beyond their professional duties, and was manifested in a willingness to perform their jobs well and to adopt an engaged approach towards the persons in their care. They also recognized the needs and security of informal caregivers as equally important to those of the person receiving care, emphasizing the significance of interaction with informal caregivers to achieve optimal care and support. The care professionals proactively sought to create a meaningful everyday life for the persons by organizing various activities such as walks, music sessions, barbecues, and baking. It was also considered essential that the persons participated in their meals, such as spreading their own sandwiches or choosing their food. Care professionals focused on the persons’ life stories, interests, and preferences to tailor activities to them.

Perhaps a group could be created. Are there others with the same interests? What can we do with this to also activate them? Because I mean, even those who do not have dementia still need to be activated and have a social context. (P 14)

Through their engagement and activities, care professionals aimed to maintain a sense of normalcy and independence as well as to create a social context and companionship among the persons.

And I’m thinking about what I’ve heard over the years, that it doesn’t just become a storage place. That there is some dignity. Security, dignity, and a reason for the persons to be there, other than that the informal caregiver gets some rest. (P 16)

That you try to create something they look forward to. That they look forward to meeting other individuals who will be there the same week. That they feel satisfied being here. To have activities with others, to socialize with someone other than the one they have at home. (P 13)

The participants described how the ***knowledge and competence*** (30) of the care professionals were fundamental in meeting the needs of both the persons and informal caregivers. This included being knowledgeable about various diseases, particularly dementia, managing change and unforeseen situations, and continuously working to build a trusting relationship and create a sense of security for both the persons and informal caregivers.

… because you need to know what you’re dealing with. If you have someone with dementia, you need to have knowledge and awareness to know … yes, how should we meet this? And who is it that I’m meeting? (P 14)

… it’s important that the care professionals have a good understanding of the care. How to assist with transfers and how to help carry out a training programme. So that they have a good understanding of that, because they need to assist many different people. And it can look completely different when the person comes back next time.// … whenever it’s not a group that I go to all the time and that’s always roughly the same and I meet it every day. Then there will be higher demands on me as a professional to handle unforeseen situations. (P 16)

The knowledge and competence of the care professionals also encompassed dealing with the person’s uncertainty and resistance in a professional.

That you have to motivate them that this will go well, it will work. You are going to get help. They can feel a lot of insecurity many times. (P 15)

The participants noted that the conditions of care professionals such as ***time and organizational resources*** (8) affected the quality of care and interactions with the persons. The design of the premises, the working hours of the care professionals, and the adaptation of work routines were significant factors. Having sufficient time for each person and attending to them at the right moment was of great importance. This also concerned collaboration with other parties such as social workers involved in the assessment process for respite care and adjusting the environment to create a safe and welcoming atmosphere. The conditions of the care professionals were enhanced through continuity in the care professional team and detailed implementation plans.

And I think that if you have the same professional team working there, who also need to have knowledge and competence about dementia and can meet these persons in an environment that is more like home, then I think that respite care becomes a little better. (P 6)

So, I think it could be a reassurance, to at least come to the same staff. I believe that having continuous professional team is good, because it’s something that lacks in home care services. So, I think it can be a security, that the persons at least come to the same professionals. (P 7)

## Discussion

The exploration of quality in respite care is needed for enhancing the lived experiences of those with chronic conditions, their informal caregivers, and healthcare and social care professionals. The insights provided in the main findings contribute to the existing literature on geriatric and dementia care services and suggest multiple areas of concern that require a nuanced discussion focusing on the implications and directions for future research. The following discussion will consider the results of the study by the two main areas, the strengths and limitations of the study, conclusions, and the need for future research.

Critical incidents in respite care, as identified by our study, primarily revolve around challenges in communication, continuity of care, and environmental adequacy; critical incidents that disrupt quality respite care. The dilemma of providing quality respite care while struggling with deficiencies in communication and continuity during care transitions reported in the findings resonate with what has been documented in the literature. Poor communication during transitions in care has been associated with adverse outcomes for patients (Coleman, 2003; Naylor et al., 2011) and the logistical issues resulting from communication failures compromise the individualization of care, a core component of quality healthcare. In continuity, the findings parallel research indicating that disruptions in care routines negatively impact patient well-being (Hirschman et al., 2023). The absence of routine can be particularly disorienting for persons with cognitive impairments, such as dementia, and can exacerbate symptoms such as agitation, leading to increased distress (Fauth & Gibbons, 2014).

The dilemma of providing quality respite care while struggling with environmental issues at respite care facilities align with the principles of environmental gerontology, which emphasizes the importance of the environment in the health and well-being of older adults (Wahl et al., 2012). It is also significant to acknowledge that the processes of belonging increase in importance as people enter advanced old age; consistent with models of human development and ageing (Tornstam, 2005). It explains why very old adults are hesitant to undertake repeated relocations; show high stability and regularity in their out-of-home related activities (e.g., preferred places and travel patterns), and value their familiar home and neighbourhood environment, even if they present inherent risks. Research also has shown that environments that are not age-friendly or dementia-friendly can exacerbate anxiety and disorientation. A high turnover and impersonal spaces may not only contribute to discomfort but also to a decline in cognitive function (Fleming et al., 2020). The identification of specific environmental shortcomings is a strength of this study. However, individual differences in environmental preferences and the potential adaptability of the persons in respite care were not explored.

The dilemma of providing quality respite care while struggling with the lack of recuperation, security and support for informal caregivers echo the substantial body of research indicating that caregiver burden is a significant issue (Adelman et al., 2014; Chiao et al., 2015; Ploeg et al., 2017). The emotional and physical toll of informal caregiving is well-documented, and respite care is intended to alleviate some of this burden. However, when respite care fails to provide relief, the benefits are negated.

The study’s insights into the nuances of caregiver burden, such as the guilt associated with respite care placement, provide valuable perspectives on strategies to alleviate the guilt, such as offering informal caregivers consultation and advice on how to manage the person’s varying preferences and ensuring that decisions about respite care are individualized to the specific needs of the person and their informal caregivers. It is important for informal caregivers to recognize that using respite care is not a sign of failure or neglect on their behalf, but a necessary step to ensure their own well-being. Gaugler et al. (2007) found that informal caregivers who experienced higher levels of guilt and burden during the transition to respite care were more likely to experience negative outcomes, such as depression and anxiety. A review by Thompson et al. (2007) found that interventions that provide information, support, and skills training can help reduce caregiver burden and feelings of guilt associated with respite care placement. It is thus suggested for future research to develop and test targeted interventions aimed at reducing informal caregiver stress, guilt and burden during transitions in care to improve their well-being.

The dilemma of providing quality respite care while struggling with inadequacies in decisions about respite care are consistent with previous literature that stresses the importance of person-centred care (PCC) and the pitfalls of a one-size-fits-all approach (Edvardsson et al., 2008; McCormack et al., 2012). The present study’s professionals’ experiences of “until further notice” decisions and lack of individualization suggest a systemic issue that merits further investigation. Financial incentives influencing care decisions have been a contentious issue in healthcare ethics and policy, which suggests a need for policy changes that allow for more flexible, personalized care decisions and a shift from financial considerations to person-centred outcomes.

The study’s proposed strategies align with the PCC paradigm, which has been advocated to improve care quality (Brooker, 2004; Santana et al., 2020). A PCC requires healthcare organizations and professionals to actively understand what the person in care needs and values. It suggests that methods for gaining this understanding need to be more widely adopted (Edgman-Levitan & Schoenbaum, 2021). There is a need to establish structured communication channels between all parties involved in the care process, ensuring that information is seamlessly transferred and accessible. Regular briefings and debriefings could be instrumental in this context, providing clarity and continuity. Personalizing the environment and tailoring activities to individual habits are practices that have been shown to improve the quality of life for those in care settings (Chaudhury et al., 2018). This could involve simple changes such as personalized rooms, homelike and comforting environment or more complex structural modifications that promote safety and mobility. Despite numerous interventions, a lack of pervasive PCC persists, and it will likely continue to be a secondary feature until it is widely understood as central to care quality (Rosvold Berntsen et al., 2021), perhaps this is due to the absence of standardized mechanisms to measure and monitor PCC at the healthcare system level and suggests that the use of metrics to measure PCC can drive changes needed to improve healthcare quality that is person-centred (Santana et al., 2020). Furthermore, we acknowledge a need to uphold the knowledge and competence of the care professionals through implementing ongoing training programs that focus on the nuances of quality respite care. This training should include strategies for handling high-stress situations and techniques for engaging with persons and their informal caregivers to understand their individual needs better.

### Strengths and Limitations

The study has several strengths that contribute to its value and relevance in the field of respite care. The strengths are highlighted by its specific focus on transition points, the preparedness of professionals, and the adaptation of work routines, shedding light on intricate aspects of respite care. The strategies align with the person-centred perspective in healthcare, emphasizing the importance of individualized care tailored to the unique needs and preferences of each person. The use of the CIT and group interviews served to collect rich, detailed data and CIT is especially suitable in exploring real situations; its strength lies in its participant-driven approach, where data collection is based on the perspectives of the participants, without forcing them into any predetermined framework (Butterfield et al., 2005). This qualitative design, underscored by an inductive research process, enabled the capture of nuanced experiences and insights into professionals’ experiences, from environmental aesthetics to the complexities of caregiving dynamics, in respite care.

However, the study is not without its limitations. The CIT requires a clear definition of what constitutes a critical incident, necessitating clarity from both the participant and the researcher about the focus of the exploration (Flanagan, 1954; Sharoff, 2008). A notable limitation is the potential for memory/recall bias in the professionals’ descriptions of incidents that happened recently, as these are easier to recall, leading to unreported or selectively reported incidents (Sharoff, 2008). The limited sample size, lack of diversity in terms of gender and experience, and the study’s geographic specificity raise questions about the transferability of the findings to other populations or healthcare systems. A limitation is also the study’s failure to fully explore the structural and systemic reasons behind the deficiencies in respite care, such as staffing constraints or policy gaps, which are essential for understanding and addressing the challenges in this field comprehensively.

## Conclusions

The study identifies the dilemma of providing quality respite care while struggling with several key barriers to quality respite care, such as communication challenges, resource constraints and emotional strain. These challenges not only impede the provision of effective respite care but also adversely affect the well-being of persons receiving care and their informal caregivers. Additionally, the study suggests strategies like improved training and support for informal caregivers, which are crucial for elevating care quality and positively impacting the experiences of all involved parties. Ultimately, the study emphasizes the need for focused efforts on communication, continuity, and a supportive environment in respite care, underlining the growing importance of this sector as the population ages. Addressing these identified challenges and applying the suggested strategies will be key to maximizing the potential of respite care as a vital support for the person receiving care and their informal caregiver.

### Future research

The study provides a sound basis for the development of interventions aimed at improving respite care. However, it does not present an implementation framework for these strategies. Future research in respite care should focus on developing interventions to address identified gaps, conducting extensive quantitative research, and examining the effectiveness of personalized care plans, improved communication, and better care environments. Studies should also investigate decision-making processes in transitioning to respite care, particularly for individuals with advanced dementia. To ensure comprehensive findings, diverse participant samples and multiple data collection methods should be used. Addressing potential limitations such as recall bias, lack of quantifiable data, and a deeper examination of economic, organizational, and policy factors is crucial for more effective policies and interventions.

## Data Availability

Data are available upon request.

## Appendix

### Interview questions used in the group interviews:

1. What constitutes quality respite care?
2. What are the conditions for experiencing safety and security for persons and their informal caregivers?
3. What are the conditions for experiencing life quality and dignity for persons and their informal caregivers?
4. Which care interventions need to be developed to enhance life quality and dignity for persons and their informal caregivers?
5. What critical incidents/dilemmas/events/situations have you encountered in respite care, and how did you resolve them?
6. What is needed to improve the situation for persons and their informal caregivers, who needs to be involved; and what is the absolute most important thing?
